# 4.5-Year Follow-up with a Novel Corneal Endothelial Prosthesis

**DOI:** 10.1055/a-2479-8606

**Published:** 2025-01-13

**Authors:** Ruth Lapid-Gortzak, Ivanka van der Meulen

**Affiliations:** Ophthalmology, Amsterdam UMC, Amsterdam, Netherlands

**Keywords:** keratoplasty, endothelial keratoplasty, corneal edema, EndoArt, keratoprosthesis, Hornhauttransplantation, Endotheltransplantation, Korneaödem, EndoArt, Korneaprothese

## Abstract

**Background** Refractory corneal edema is the foremost reason for endothelial corneal transplantation (EK) in the world. Descemet membrane endothelial keratoplasty (DMEK) and Descemet stripping automated endothelial keratoplasty (DSAEK) offer good clinical outcomes. However, human donor tissue is limited in availability and has a complex logistical chain. Many patients in need do not have access to EK. Success of the human donor tissue depends on its quality, the number of rebubbles, repeat keratoplasties, and patient-related risk factors. The use of an endothelial corneal keratoprosthesis could benefit patients with a high risk for graft failure and human graft rejection, and patients in underserved areas where tissue banking is unavailable.

**Patients and Methods** Case cohort series of 10 eyes of 10 patients implanted with the EndoArt, with a follow-up of up to 4.5 years. Outcome measures are device adherence, central corneal thickness, visual acuity, and incidence of adverse events.

**Results** In the first seven eyes, the EndoArt was implanted in eyes with low visual potential within the first-in-human (FIH) trial, and in the three remaining eyes, implantation was performed because of either guarded posterior or anterior segment prognosis or the patientʼs wishes. In 9/10 eyes, device adherence was obtained and maintained throughout the follow-up period. Mean corneal thickness decreased from 927 + SD 241 microns to 621 + SD 140 microns. In 6/10 eyes, visual acuity improved from a mean preoperative value of logMAR 1.94 + 0.63 to a mean postoperative value of logMAR 0.8 + 0.64. No pathological thinning or corneal metabolic changes were seen. The surgical technique was developed during the follow-up period.

**Conclusion** The EndoArt provided a stable decrease in central corneal thickness, improved vision in 60% of eyes, and had no device-related adverse events. The EndoArt is an additional treatment modality for special cases like repeat corneal graft failure in the population of the Western world, or as a primary procedure in underserved areas worldwide.

## Zusammenfassung

**Hintergrund** Das refraktäre Hornhautödem ist die Hauptursache für eine endotheliale Hornhauttransplantation (EK) in der Welt. Descemet-Membran-Endothel-Keratoplastik (DMEK) und Descemet Stripping Automatisierte Endothelkeratoplastik (DSAEK) bieten gute klinische Ergebnisse. Menschliches Gewebe ist in seiner Verfügbarkeit begrenzt und hat eine komplexe logistische Kette. Viele bedürftige Patienten haben keinen Zugang zur EK. Der Erfolg des menschlichen Spendergewebes ist Abhängig von der Qualität des Gewebes, von der Anzahl des Re-Bubblings, von wiederholten Keratoplastiken und patientenbezogenen Risikofaktoren. Die Verwendung einer endothelialen Hornhautkeratoprothese könnte Patienten zugute kommen, die ein hohes Risiko für ein Transplantatversagen und Abstoßung des menschlichen Transplantats haben, sowie Patienten in unterversorgten Gebieten, in denen keine Gewebebanken zur Verfügung stehen.

**Patienten und Methoden** Fallkohortenserie von 10 Augen von 10 Patienten, denen die EndoArt implantiert wurde, mit einer Nachbeobachtung von bis zu 4,5 Jahren. Ergebnismaßstäbe sind die Geräteeinhaltung, die Dicke der zentralen Hornhaut, die Sehschärfe und die Inzidenz von unerwünschten Ereignissen.

**Ergebnisse** Bei den ersten 7 Augen wurde die EndoArt in Augen mit geringem Sehpotenzial innerhalb der First-in-Human-Studie (FIH) implantiert. In den 3 verbliebenen Augen wurde die Implantation entweder aufgrund einer unsicheren Prognose des hinteren oder vorderen Augenabschnitts oder aufgrund des Wunsches des Patienten durchgeführt. In 9/10 Augen wurde die Geräteeinhaltung erreicht und während des gesamten Nachbeobachtungszeitraums aufrechterhalten. Die mittlere Hornhautdicke verringerte sich von 927 ± SD 241 Mikrometer bis 621 ± SD 140 Mikrometer. In 6/10 Augen gab es eine Verbesserung der Sehschärfe gegenüber dem mittleren präoperativen Wert von logMAR 1,94 ± 0,63 auf einen mittleren postoperativen Wert von logMAR 0.8 ± 0.64. Eine krankhafte Ausdünnung oder Hornhautverstoffwechselung waren nicht zu beobachten. Die Operationstechnik wurde während der Nachbeobachtungszeit entwickelt.

**Schlussfolgerung** Die EndoArt führte zu einem stabilen Rückgang der zentralen Dicke der Hornhaut, verbesserte die Sehkraft bei 60% der Augen und führte zu keinen produktbedingten unerwünschten Ereignissen. Die EndoArt ist eine zusätzliche Behandlungsoption für Sonderfälle, wie wiederholtes Hornhauttransplantatversagen in der Bevölkerung der westlichen Welt oder als primäre Methode in unterversorgten Gebieten weltweit.

## Introduction

Corneal edema is the final common pathway for endothelial failure. Causes for endothelial failure can be from dystrophies, or iatrogenic, surgical trauma from cataract surgery, glaucoma shunting surgeries, silicon oil after vitrectomy surgeries, and previous corneal transplantation, either endothelial or full thickness. Corneal edema is the main indication for endothelial keratoplasty. Until recently, the only way to treat corneal edema was performing a form of keratoplasty with human corneal donor tissue. The practice patterns differentiate in geography, as in the Western world, endothelial keratoplasties are the leading treatment, and in the rest of the world, if tissue is available at all, it is a penetrating keratoplasty 1 , 2 , 3 , 4 .

Using an artificial endothelial barrier to treat corneal edema has been tried in the past 5 , 6 , 7 . However, these previous attempts were unsuccessful. Recently, a corneal endothelial prosthetic device, the EndoArt (EyeYon Medical, Nes Ziona, Israel), has been registered and shown to be safe in the first-in-human (FIH) trial. In that trial, safety and adherence of the device during the first year post-implantation was proven 8 . It has been shown that the EndoArt decreases central corneal thickness (CCT) and improves cornea clarity after implantation 9 .

The EndoArt was invented by Offer Daphna after observing that aphakic eyes filled with silicon oil exhibited clear corneas if the silicon oil was in place, despite low endothelial cell counts. It is thought that silicone oil allows for a barrier mechanism, conveying a steady state between inflow and outflow of fluids into the cornea. Immediately after removing the silicon oil corneal edema ensued in those eyes 10 . In healthy corneas, the endothelium has a pump function that determines cornea thickness and clarity. In corneal endothelial disease states, corneal thickness and clarity are compromised. The fluid balance in the cornea determines the corneal thickness 11 .

The EndoArt is a 6.5-mm dome-shaped 50-micron thick acrylic implant that functions as a barrier when placed against the endothelial side of the cornea, allowing for clearing of the cornea from excessive fluid and leading to a new steady state and decreased corneal thickness. However, long-term results need to be reported.

We report the outcomes of up to 4.5 years of our cohort at the Amsterdam University Medical Centers.

## Patients and Methods

*Patient population* . Our cohort comprises of seven eyes of seven patients who were included in the FIH study. All eyes had cornea edema, without previous corneal or glaucoma surgery, extensive posterior segment pathology, and a bad visual prognosis. The other three eyes of three patients were implanted after the EndoArt became available as a treatment option. These patients either wanted to have the artificial corneal prosthesis because of personal motivation or had extensive anterior segment pathology, including repeated graft failures, glaucoma implants, trauma, and other pathologies, and were not deemed good candidates for human donor tissue grafting of the cornea.

*EndoArt* . The EndoArt has an “F” stamp that guides the directionality of implantation. It has a rim that needs to be snug opposed to the posterior cornea during implantation.

*Surgical procedure* . The surgical technique was developed during the FIH study. At first, rebubbling rates were high, and in our 4th case, Dr. Lapid-Gortzak decided upon suturing of the EndoArt. The suture prevented further rebubblings and was removed 4 weeks after implantation. Nowadays, suturing has become the standard of care. Implantation was done with a medical cartridge, and later with forceps, but as more people use the EndoArt, more experience is gained and surgeons have also inserted the EndoArt on top of a blunt spatula, with good results.

### Case Series

The first seven cases are from the FIH study and the last three cases are not.

#### Case 1

**Fig. 1:**
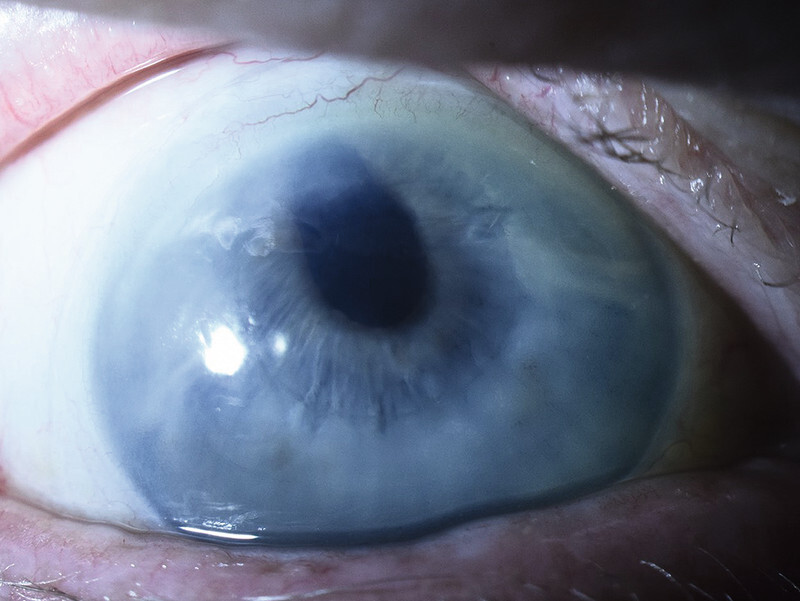
The EndoArt adherent for 3.5 years with a central corneal clarity allowing for a visual acuity of 0.4. Patient has complex posterior segment pathology limiting visual acuity (patient 1).

An 82-year-old patient presented after complicated cataract surgery with vitrectomy and Artisan (Artisan aphakia lens, Ophtec, Groningen, The Netherlands) implantation as well as geographic macular atrophy. EndoArt implantation was done successfully, and no suture was placed. After two rebubble procedures, the EndoArt has been in place for nearly 4 years. Maximal visual acuity was logMAR 0.4 after EndoArt placement ( Fig. 1 ). At the last visit, the patient had a retinal detachment, and even though the VR surgeons offered surgery, the patient declined. The cornea and EndoArt were clear enough to perform vitreoretinal surgery.

#### Case 2

A 71-year-old patient presented after angle closure glaucoma and complicated cataract surgery in nanophthalmos. Because of end-stage glaucoma, the eye was deemed to have no visual prognosis. EndoArt was implanted without sutures. After three rebubbles, the EndoArt remained adherent. The cornea cleared up centrally, but not peripherally. The patient did not gain visual acuity, as was expected. No adverse events were seen.

#### Case 3

A 60-year-old patient presented after phakic Artisan for hypermetropia, pseudophakic bullous keratopathy (PBK), complicated Artisan removal surgery, cataract surgery, and glaucoma. EndoArt implantation was uneventful, and the EndoArt remained adherent after one rebubble. The best postoperative visual acuity was 0.5. After this, bullae developed, which were treated with anterior stromal needling, to no avail. The patient was lost to follow-up at the end of the FIH study.

#### Case 4

A 57-year-old patient presented with constitutional atopia, complicated cataract surgery, Artisan implantation, postoperative uveitis, posterior vitrectomy, cystoid macular edema (CME), and chronic CME. The EndoArt was implanted and rebubbled five times. We are unsure whether eye rubbing may have played a role in detaching the EndoArt. At the 6th rebubble, it was decided to suture the EndoArt. This is the first time a suture was used on the EndoArt. The EndoArt has remained in place ever since. The suture was removed after 4 weeks. The patient had herpes stromal keratitis after 1.5 years with local thinning in that area, which was outside the diameter of the EndoArt. To avoid the risk of thinning with topical steroid therapy, an oral prednisone pulse course of 40 mg was administered for 3 days, with half doses and stopping in 12 days. The stromal keratitis responded well. Since then, three recurrences have occurred, all responding to this treatment. The corneal thinnest point fluctuated between 444 microns and 490 microns in topography.

#### Case 5

A 67-year-old patient was referred after a Descemet removal, the only procedure in a highly myopic, deeply amblyopic eye with Fuchs endothelial dystrophy. The patient underwent an uncomplicated EndoArt implantation with a decrease in CCT from 825 microns to 633 microns. No rebubble was needed, and one suture was used. Peripheral bullae were treated with a bandage contact lens for 2 years. Since, no new bullae formation was seen, and the central cornea has remained clear. No adverse events were seen.

#### Case 6

A 62-year-old patient presented with a history of high myopia, trauma, retinal detachment, posterior vitrectomies, and pseudophakia. After uneventful EndoArt implantation with one suture and no rebubble, CCT decreased from 766 microns to 571 microns. This patient gained vision from logMAR 1.3 to logMAR 0.8 (decimal 0.2). No adverse events were seen.

#### Case 7

A 70-year-old patient presented after retinal detachment, posterior vitrectomy, and macular scarring. EndoArt implantation performed with one rebubble, and one suture. Visual acuity improved from logMAR 2 to logMAR 0.9 (decimal 0.16), and CCT decreased from 869 to 573 microns. The EndoArt has been adherent, and no adverse events were seen.

#### Case 8

A 69-year-old patient presented with keratoconus after perforating keratoplasty, DSEK, DMEK, Baerveld implants, and repeated rejections. EndoArt implantation on the posterior irregular surface of the penetrating keratoplasty (PKP) was difficult. Three attempts at surgery and four sutures were used to try and obtain a 360-degree adherence of the EndoArt rim. With partial adherence, a decrease in CCT from 1322 to 1080 microns was achieved. At the 6-month follow-up, we advised the patient to undergo a re-PKP. This is the only case in which we failed to achieve adherence.

#### Case 9

An 87-year-old patient presented after PBK, chronic cystoid macula edema and posterior uveitis, and a failed DSAEK, and underwent an uneventful EndoArt implantation with one suture. One rebubble with the addition of a second suture was done, and the EndoArt has remained in place. CCT decreased from 724 to 432. Visual acuity improved from logMAR 2.18 to logMAR 0.7 (decimal 0.3). No adverse events were seen.

#### Case 10

**Fig. 2:**
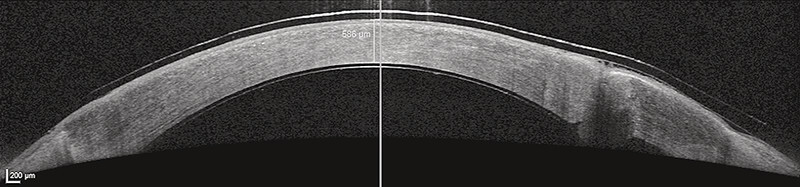
Optical coherence tomography of a successfully adherent EndoArt in a post-PKP eye (patient 10).

A 58-year-old patient presented after penetrating trauma, subluxated intraocular lens (IOL) after repeated surgeries, sulcus IOL, posterior vitrectomy, and limbal stem cell deficiency. EndoArt was implanted for painful bullous keratopathy. At first surgery, initial adherence was achieved with one suture. The nasal and temporal edges of the PKP were very fibrotic, and a scar on the endothelia side of the nasal PKP was removed with microforceps. Two rebubbles were performed, with suture adjustment. Finally, with four sutures, good 360-degree adherence of the EndoArt was achieved. The CCT fluctuated from no EndoArt 730 microns to initial attachment of 586 microns, to detachment of 730 microns, and final attachment and adherence of 490 microns. The cornea cleared up ( Fig. 2 ). Intraoperative epithelium removal lead to a persisting epithelial defect, which took 1 month to heal with serum drops. The patient did not gain visual acuity, as expected.

**Fig. 3:**
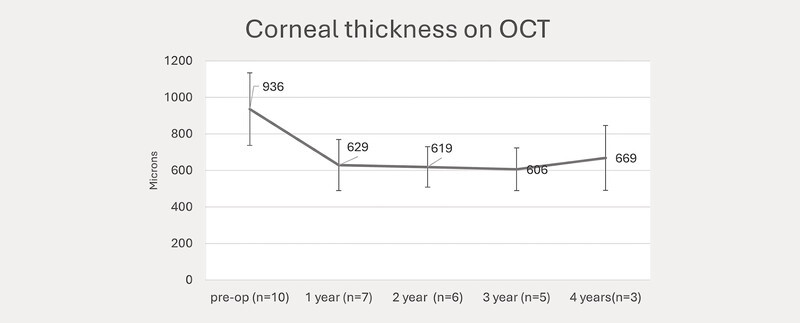
Graph showing the mean central corneal thickness over time.

**Fig. 4:**
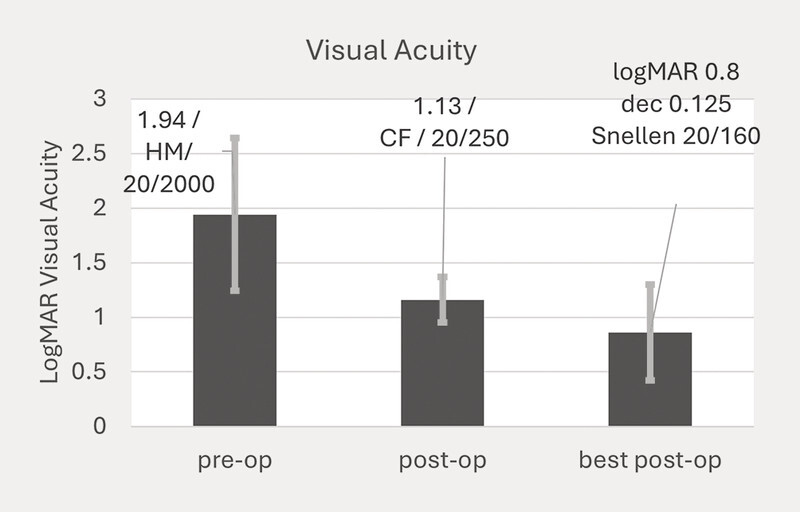
Mean visual acuity from preoperative to postoperative and best-case scenario.

The numerical demographic data of the cases, number of rebubbles and sutures, and the change in CCT and visual acuity are summarized in Tables 1 , 2 , 3 and Figs. 3 and 4 . CCT decreased from a mean preoperative 927 ± SD 241 microns to 621 ± SD 140 microns ( Fig. 3 ). Visual acuity improved as a mean from logMAR 1.94 mean ± 0.64 SD to 0.8 mean ± 0.64 SD (see Fig. 4 ).

**Table:** **Table 1** Central corneal thickness data from preop to postop.

Patient	Preop (microns)	Last postop (microns)	1 year (microns)	2 years (microns)	3 years (microns)	4 years (microns)
1	1 087	738	728	680	662	662
2	827	868	838	811	796	850
3	839	680	743			
4	854	469	451	530	444	489
5	1 135	786	569	514	590	
6	766	548	548	587	571	
7	869	556	530	593	573	
8	1 322	1 261				
9	724	530				
10	730	490				
Average	936	692.6	629	619	606	669
SD	199	241	140	111	117	178

**Table:** **Table 2** Rebubble rates and sutures used in each case.

Patient	1	2	3	4	5	6	7	8	9	10
Total rebubble	2	3	1	6	0	0	1	3	1	2
Total sutures	0	0	0	1	1	1	1	4	2	4
Success/fail	success	success	success	success	success	success	success	fail	success	success

**Table:** **Table 3** Visual acuity results in each case.

Patient	Preop	Postop best	Postop last
1	2	0.3	0.82
2	2	2	2
3	0.6	0.3	2
4	2	1.3	1.3
5	3	1.3	1.3
6	1.3	0.8	0.8
7	2	0.8	0.8
8	2.18	2.18	2.18
9	2.18	0.7	0.7
10	2.47	1	1.1
Avg. logMAR	1.97	1.68	1.3
SD	0.643 636	0.63 911	0.566 157

## Conclusion

This report shows the results of the EndoArt implantations performed at our center in the past 5 years. The EndoArt was invented after observing that patients with silicone oil in their eye had a perfectly clear cornea, until the silicone oil was removed. The EndoArt has been shown to adhere and allow for a new status quo in the water balance in the cornea, with a clearing up of the cornea and a decrease in CCT 8 , 9 .

In seven patients, the EndoArt was implanted within the framework of the FIH study and the subsequent patients were implanted for complex indications: repeated failed grafts with poor prognosis. In our cohort, we see that successful adherence of the EndoArt was achieved in 90% of patients. An improvement in visual acuity was also achieved in very difficult eyes, but no good functional vision was obtained because of the complex initial pathology. Fontana et al. have shown good results in terms of visual acuity, with patients achieving 0.3 logMAR visual acuity 12 .

We have shown that adding a suture to the surgery protocol increases adherence of the EndoArt and decreases rebubble rates in eyes with pristine corneas. However, this is not true for corneas with complex posterior surfaces, like post-PKP eyes. The decrease in CCT was best in eyes that did not have massive corneal edema preoperatively. In one eye with an axial length of 38 mm and a white-to-white in excess of 12.5 mm, the result of corneal clearing was less. This leads to the question whether sizing of the implant should maybe be stratified to ocular measurements to prevent over- or undercorrection of corneal desiccation.

The use of an endothelial barrier has been shown in clinical practice in aphakic eyes filled with silicone oil, and the rapid failure of the endothelium once the silicone oil is removed from such eyes 10 . Artificial implants have not worked in the past, probably because of material and design issues. The use of an anchoring suture, or now preferably up to three anchoring sutures, makes the procedure more viable, with better adherence and less rebubbling. We have also shown that adherence of the EndoArt reduces corneal thickness, and that detachment causes corneal thickening again, which is alleviated by reattachment (patient 10). Clinical effect of adherence is directly related to corneal thickness. Failure of adherence occurred in one patient, who had repeated PKPs and other corneal grafts, and had very advanced corneal edema, with a preoperative corneal thickness of 1322 microns. Implementing the novel technology in difficult cases will contribute to the learning curve and limit the later indications for the use of an EndoArt for cases in which the prognosis will be more predictable.

Currently, with a higher demand for human corneal graft tissue in the world than availability 13 , an artificial implant with a cheaper storage solution – i.e., devices packaged off the shelf instead of a tissue bank, good availability, scalability to all parts of the world – will allow for a more sustainable treatment of corneal blindness from corneal edema and endothelial failure. The EndoArt can also be used as a temporizing measure in case this is deemed beneficial to the patient. Indication for implantation will vary geographically, with a place for the EndoArt being reserved for the more complex cases in the Western world where tissue availability is less of a problem to a go-to technique in countries where there is less tissue availability and tissue banking options. The EndoArt in this will contribute to democratization and equity in terms of corneal transplantation care around the globe.

The limitation of our report is the fact that we have done a limited number of cases, some with limited follow-up periods. Our caseload is relatively complex. Also, the surgical procedure was still under development and not yet standardized. On the other hand, it is important to share as much data as possible to contribute to a robust data set in the literature on this novel technology. In our cohort, we found an improved visual acuity in most patients.

A novel endothelial prosthetic device such as the EndoArt has a 90% chance of successful long-term and short-term adherence in a variety of complex pathologies. The EndoArt leads to clearing up of the central cornea, with improved visual acuity in many cases. No pathological thinning was seen in any of the eyes. More extensive follow-up and enlargement of reported cohorts are needed to validate the efficacy of this technique in the treatment of corneal edema.
